# Spreading activation in nonverbal memory networks

**DOI:** 10.1007/s40708-016-0058-y

**Published:** 2016-11-28

**Authors:** Paul S. Foster, Candias Wakefield, Scott Pryjmak, Katelyn M. Roosa, Kaylei K. Branch, Valeria Drago, David W. Harrison, Ronald Ruff

**Affiliations:** 1 Middle Tennessee State University, Murfreesboro, TN USA; 2 University of Florida, Gainesville, FL USA; 3 UOC Neurologia, ASP Siracusa, Ospedale “Muscatello” Augusta, Syracuse, Italy; 4 Behavioral Neuroscience Laboratory, Psychology Department, College of Science, Virginia Polytechnic Institute, Blacksburg, VA 24061-0436 USA; 5 San Francisco Clinical Neurosciences and University of California, San Francisco, USA

**Keywords:** Spreading activation, Memory, Visuospatial, Right hemisphere, Verbal fluency, Design fluency, Frontal lobe, Neuropsychological tests

## Abstract

Theories of spreading activation primarily involve semantic memory networks. However, the existence of separate verbal and visuospatial memory networks suggests that spreading activation may also occur in visuospatial memory networks. The purpose of the present investigation was to explore this possibility. Specifically, this study sought to create and describe the design frequency corpus and to determine whether this measure of visuospatial spreading activation was related to right hemisphere functioning and spreading activation in verbal memory networks. We used word frequencies taken from the Controlled Oral Word Association Test and design frequencies taken from the Ruff Figural Fluency Test as measures of verbal and visuospatial spreading activation, respectively. Average word and design frequencies were then correlated with measures of left and right cerebral functioning. The results indicated that a significant relationship exists between performance on a test of right posterior functioning (Block Design) and design frequency. A significant negative relationship also exists between spreading activation in semantic memory networks and design frequency. Based on our findings, the hypotheses were supported. Further research will need to be conducted to examine whether spreading activation exists in visuospatial memory networks as well as the parameters that might modulate this spreading activation, such as the influence of neurotransmitters.

## Introduction

Collins and Loftus [1] proposed a model of spreading activation in semantic networks stating that specific semantic memories (e.g., raspberries) are organized into a larger network that comprises concepts (e.g., fruit). Semantic memories are then represented as nodes within these conceptual networks, and the semantic nodes (e.g., raspberry and strawberry) within a conceptual network (e.g., fruit) are more strongly interconnected through associative, bi-directional links than are semantic nodes from different conceptual networks (e.g., raspberry and automobile). The strength of the associative, bi-directional links varies between nodes within a conceptual network, with some connections being quite strong (e.g., raspberry and blackberry) and others being relatively weaker (raspberry and orange). Activation of any given node will spread along associative links to other related nodes that also comprise the network, spreading first to the nodes linked to the first node and then to the nodes that are linked to each of these and so forth. The strength of the connections between conceptual nodes is partially determined by production frequency norms or the frequency of use of the links.

Further, the strength of the connectivity between conceptual nodes is likely related to the Hebbian principle that neurons (or neuronal assemblies that comprise the semantic network) that fire together wire together ([2, p. 70]; see also [3, 4]). The speed of spreading activation is determined by the strength of the associative links between the nodes. The extent or spread of activation is partially dependent on the strength of the initial activation of the node, such that greater initial activation will result in greater spread of activation from that node. Hence, greater initial activation will result in a greater spread of activation from that initially activated node to other nodes, including those with weaker direct or indirect connections to the initially activated node. The spreading of activation then decreases over time and/or some intervening activity.

There have been a number of other models of spreading activation and semantic priming proposed in the literature (for review see [5]). These models all share the common feature of addressing the structure of, and the flow of, information within semantic memory. As such, these models all involve verbal memory networks. However, research has indicated that separate verbal and nonverbal or visuospatial memory networks exist and neuropsychological efforts frequently employ double dissociation techniques between these systems. A number of factor analytic studies have been conducted to investigate material-specific memory. Although the results of these investigations have been far from unequivocal, the results of many support the existence of separate verbal and visuospatial memory systems. For instance, separate verbal and visuospatial memory factors for the Wechsler Memory Scale—Revised have been reported in normal individuals [6, 7]. Other studies using a variety of tests of verbal and visuospatial memory within a larger battery of neuropsychological tests have also found separate factors [8–11].

Differential involvement of the left and right hemispheres in verbal and visuospatial memory has also been reported (see [12]). Whereas verbal memory is associated with left hippocampal volume, visuospatial memory is associated with right hippocampal volume [13], although not all studies find this relationship. Electrical stimulation of the left hippocampus results in impairment in the recognition of words and stimulation of the right hippocampus impairs recognition for faces [14]. Encoding of words is associated with left hippocampal activation and encoding of faces is associated with right hippocampal and amygdala activation [15]. Others have found that left temporal lobe epilepsy patients evidence relative impairment in verbal memory and right temporal lobe epilepsy patients evidence impairment in visuospatial memory [16–20]. Graydon et al. [21] found a decline in auditory verbal memory following left unilateral temporal lobectomy and a decline in visuospatial memory following right unilateral lobectomy. Similar findings have been reported by Pillon et al. [22] who demonstrated double dissociation between patients with right or left temporal lobectomies and performance on visuospatial versus auditory verbal memory tasks, respectively.

Given the existence of separate verbal and visuospatial memory networks, the possibility exists that spreading activation may also occur in visuospatial memory networks. This hypothetical network may have comprised the lines, angles, and shapes to which we are exposed and which comprise the objects that are stored in memory. Hence, in so much as a network of verbal memories exists throughout which a spread of activation flows from one conceptual node to another, then a similar spread of activation might exist in a visuospatial memory network. Considerable overlap may exist between verbal and visuospatial memory networks, in as much as the shapes of objects are stored in a visuospatial memory network and these objects are associated with verbal labels that are stored in semantic/conceptual networks. However, the possibility also exists that a visuospatial memory network exists that is separate from the verbal memory network. Establishing the possibility that a visuospatial memory network exists, though, creates the unique challenge of measuring, manipulating, and investigating this hypothetical network.

Semantic priming paradigms, such as lexical decision tasks, have been a primary method of investigating spreading activation in lexical/semantic memory networks. Lexical decision tasks involve presenting word pairs in a successive fashion with the individual being asked to indicate whether the target stimulus is a real word or a nonsense string of letters. Additionally, the target word is sometimes preceded by a semantically related prime word (e.g., cat–tiger) and at other times the target word is preceded by an unrelated prime word (e.g., screwdriver–tiger). Typically, the reaction time for the related pairs is significantly faster than the reaction time for the unrelated pairs. This finding indicates that the related prime word activated the semantic network associated with that word and hence facilitated judgment on whether the target word is a real word or not.

Priming tasks have also been used to investigate implicit memory for visual objects. The priming paradigm used in these investigations involves presenting three-dimensional objects, some of which are structurally possible and some of which are not structurally possible; in that they contain inconsistencies in surface structure that would preclude them from existing as actual three-dimensional objects. Implicit memory is then assessed with a priming task involving the brief presentation of objects that were presented and other objects that have not been previously seen. The task for the participant is to decide if the object presented is possible or impossible. Priming is then indicated by more accurate decisions about objects that have been previously presented as compared to objects that have not. Using this paradigm, Schacter and colleagues have demonstrated no significant differences in priming between older and younger adults, despite older adults having worse explicit memory for the objects [23]. Also, transformations of the size and reflection of the objects do not seem to affect priming for objects, even though these transformations do affect explicit memory for the objects [24]. Patients with impaired explicit memory functioning from cerebrovascular disorders exhibit priming of objects as well [25]. The results of studies conducted by Schacter and colleagues have supported the contention that structural information about objects is represented in an implicit memory system that is separate from the explicit memory system in which object meaning is stored [26].

Although the priming paradigm used by Schacter and colleagues may be used to investigate implicit memory for visual objects, this paradigm is not very amenable for investigating whether spreading activation may exist in visuospatial memory networks. Investigating spreading activation in visuospatial memory networks would require the stimuli in this priming paradigm to have some meaningful relationship, something akin to the cat–tiger relationship used in lexical decision tasks. To investigate spreading activation in semantic memory networks, some of the stimulus words have an association with the target words, coming from the same semantic network. However, the strength of the bi-directional associations within a conceptual network cannot be manipulated when the stimuli comprise nonsense three-dimensional figures. Hence, a method for measuring spreading activation within visuospatial memory networks needs to be developed.

We have used a completely different paradigm to investigate spreading activation in lexical and semantic memory networks. Our approach to measuring spreading activation is based on the responses from the Controlled Oral Word Association Test (COWAT) and Animal Naming (AN) test. The COWAT requires the individual to generate as many words as possible that begin with a specified letter in 60 s and the AN test requires the individual to name as many animals as possible within 60 s. Measuring the extent of spreading activation involves calculating the average word frequency for the words generated on the tests. Based on the aforementioned Hebbian principle, words that occur more frequently in the English language should have stronger representations and lower thresholds for activation. Further, because of production frequency norms, more frequently used words should have stronger and more numerous connections with other words in the lexical (COWAT) or semantic (AN) network. Conversely, words that occur relatively infrequently should have weaker neural representations and fewer associations with other words. Because spreading activation is associated with the frequency with which words are reported, high frequency words will elicit stronger spreading activation of corresponding neural networks. One of the results of this increase in spreading activation is that neural networks associated with lower frequency words are proposed to be activated. Hence, when computing the average word frequency for the words generated on the COWAT or on the AN test, increasing spreading activation will result in a lower overall average word frequency since a greater number of lower frequency words will be included. Additionally, decreased spreading activation will increase the average word frequency due to the inclusion of more words with higher frequencies. The frequency is determined by how often the words generated are normally used by speakers-readers of English. Support for this use of the COWAT and the AN test as an index of spreading activation is provided by the results of research using lexical decision tasks. Specifically, this research has indicated that the reaction time for identifying high-frequency words is significantly faster than for low-frequency words [27–29]. The longer reaction time for lower frequency words suggests that the adequate activation of the nodes that represent lower frequency words requires greater spreading activation. Essentially, greater spreading activation is required to activate words that have lower frequencies, i.e., are further out in the lexical or semantic network.

Using our word frequency paradigm for measuring spreading activation, we have found increased spreading activation in individuals with relatively higher scores on the Beck Depression Inventory—II [30]. Further, patients with Alzheimer’s disease exhibit increased spreading activation in lexical memory networks and decreased spreading activation in semantic memory networks [31, 32]. Patients with Parkinson’s disease (PD) were also found to exhibit increased spreading activation in lexical memory networks [33]. We have also used this paradigm to investigate the effects of acetylcholinesterase inhibitors (AChEIs) on spreading activation, finding that AChEIs have the effect of reducing spreading activation in lexical memory networks [34]. Finally, we have recently reported a significant relationship between spreading activation and memory functioning. Specifically, our findings indicated that increased spreading activation was associated with better immediate and delayed recall of a word list [31, 32].

Our word frequency paradigm represents a more “top–down” approach to measuring spreading activation by having individuals create their own stimuli and hence may also represent more of a “free” or “natural” flow of spreading activation within lexical/semantic networks. This approach to measuring spreading activation is also not as constrained by experimental methodologies as are lexical decision tasks, for which specific words are chosen by the experimenter. More importantly, for the purpose of this investigation, our use of word frequencies from the COWAT and AN test to measure spreading activation is potentially adaptable for measuring spreading activation in visuospatial memory networks. Specifically, the Ruff Figural Fluency Test (RFFT) [35] is a visuospatial analogue of the COWAT and is generally accepted as a measure of nonverbal fluency. Research has indicated that performance on the RFFT is sensitive to right frontal lobe dysfunction [36]. Additionally, we have previously reported that heightened delta EEG amplitude over the right frontal lobe is associated with reduced performance on the RFFT [37]. The use of the RFFT to investigate spreading activation in visuospatial memory networks may be accomplished in a manner similar to that of the COWAT. Specifically, the paradigm would involve calculating the frequency of each unique design produced on the RFFT, based on a large dataset from administration of the RFFT. Analogous to the COWAT, designs that are produced less frequently should be further out in the visuospatial memory network, being comprised more abstract designs for which one is not as likely to come into contact. Conversely, more frequent designs should be closer in the network and have stronger connections with other designs as these designs include figures that comprise many of the objects that we see on a daily basis, such as a simple straight line. The purposes of this investigation included constructing a design frequency corpus, determining how spreading activation in nonverbal memory networks relates to demographic variables, examining the relationship between design frequency and performance on tests of left and right hemisphere functioning, and examining the relationship between verbal and visuospatial spreading activation.

## Experiment I

We sought to describe the development of a corpus of design frequencies based on the RFFT and to explore how design frequency is related to a variety of demographic variables (sex, age, education, intelligence) and to neuropsychological tests of left and right hemisphere functioning. The Vocabulary subtest of the Wechsler Adult Intelligence Scale—Revised (WAIS-R) was used as a measure of left hemisphere functioning do to the larger body of scientific findings on this version in comparison with the WAIS-IV. Research has indicated that patients with left temporal lobe epilepsy perform worse on the Vocabulary subtest than patients with right temporal lobe epilepsy [38]. Left temporal lobe brain damage has also been associated with worse performance on the Vocabulary subtest [39]. The Block Design subtest of the WAIS-R was used as a measure thought to be relatively sensitive to right hemisphere functioning, although not exclusive to these brain regions. Research has indicated that right temporal lobe epilepsy patients perform significantly worse on the Block Design subtest than those with left temporal lobe epilepsy [40]. Right parietal lesions have also been associated with impaired performance on the Block Design subtest [41]. We also used the Finger Tapping Test to gain indications of left and right hemisphere functioning, since finger tapping is associated with contralateral activation of the sensorimotor cortex [42, 43]. Finally, the total number of unique designs produced on the RFFT was also used to determine if a relationship exists between the number of designs and the average design frequency. Our primary hypothesis was that a negative relationship would exist between design frequency and measures of right hemisphere functioning and that no relationship would exist between design frequency and measures of left hemisphere functioning.

## Methods

### Participants

Our sample consisted of 173 individuals (85 women and 88 men) from the original normative sample for the RFFT [44]. The ages of our participants ranged from 16 to 69 years (*M* = 41.36, *SD* = 15.39) and the level of education ranged from 9 to 21 years (*M* = 14.23, *SD* = 2.36). There were a total of 12 left-handed individuals and 161 right-handed individuals. The participants did not have any history of psychiatric hospitalization, chronic drug abuse, or neurological disorder.

### Apparatus

*Block Design* The Block Design (BD) subtest of the WAIS-R is a test of visuospatial ability requiring individuals to arrange 9 blocks in a particular pattern that replicates a picture that is shown to them. The blocks have some sides that are all red, some sides that are all white, and some sides that are diagonally divided between red and white. Some of the patterns use a 2 × 2 arrangement and other, more difficult patterns use a 3 × 3 arrangement of the blocks. A total of 9 different designs are presented for the individuals to reproduce. The maximum score is 51, with scores on individual items ranging from 0 to 4 and bonus points awarded for quick, perfect performance. The age-corrected Scaled Score was the variable of interest in this study.

*Design frequency corpus* The design frequency corpus (DFC) contains a corpus of 1397 different designs that were produced on the RFFT by our sample. Each design produced on the RFFT is represented in the corpus only once and the number of times (i.e., frequency) each design was produced is provided (see below for specific procedure in creating the DFC). The DFC is organized such that the frequencies of the designs produced are available for each of the three different stimulus patterns. Further, the first stimulus pattern is administered over three separate trials, with trials 2 and 3 containing background distracters. Thus, the DFC lists not only the total number of times each design was produced across the three different trials but also the total number of times each design was produced for each separate trial. The variable of interest in this study was the average design frequency for the designs produced

*Finger Tapping Test* The Finger Tapping Test (FTT) is a test of motor speed that consists of a mechanical counter mounted on a small (10″ × 10″) board and on which a specially designed arm is mounted that measures the number of oscillations of the finger. Participants are instructed to use the finger of their dominant hand (FTD) to tap the arm of the mechanical counter as fast as possible and then repeat this using the nondominant hand (FTND). The test requires five separate trials for each hand, with each trial lasting 10 s. The average of the five trials was the variable used in this study consistent with the preponderance of the literature using this measure.

**Fig. 1:**
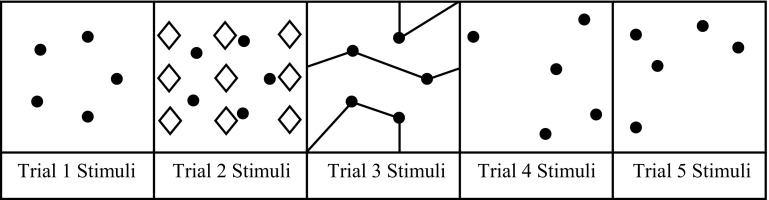
RFFT stimuli for the five different trials

*Ruff Figural Fluency Test* The Ruff Figural Fluency Test (RFFT) was administered to assess design fluency and to calculate design frequency. The RFFT [35] is a measure of nonverbal fluency consisting of five individual parts, with each part consisting of a unique stimulus pattern. More specifically, each of the five parts contains a 5 × 7 array of 35 unique stimulus items, with each stimulus item being comprised a 5-dot matrix (see Fig. 1). Additionally, each stimulus sheet is preceded by three sample stimulus items for the participant to complete. The participants were instructed to draw as many unique designs as possible by connecting two or more of the dots within each of the matrices within a time limit of 1-min. The first three trials contain the same stimulus pattern but with different distracters placed in the background. The fourth and fifth trials each contain a different 5-dot matrix. The total number of unique designs produced across the five trials was used in this study.

*Vocabulary* The Vocabulary test is a subtest of the WAIS-R that requires the individuals to orally define a series of 35 words that are presented in increasing difficulty. Each item is scored 0, 1, or 2 and the age-corrected Scaled Score was the variable of interest in this study.

*Wechsler Adult Intelligence Scale—revised* The WAIS-R is a measure of intelligence consisting of eleven subtests, including six verbal tests and five nonverbal tests. Performance on the verbal and nonverbal tests yields both a Verbal IQ and a Performance IQ, respectively. A Full Scale IQ is also derived based on overall performance. The Full Scale IQ was the variable of interest in this study.

### Procedure

This study was approved by the Middle Tennessee State University Institutional Review Board and all participants provided written informed consent. The WAIS-R, including the Vocabulary and BD subtests, was administered according to standard procedures, as were all other tests. The development of the DFC involved administering the RFFT test using standard procedures. Subsequently, each different design produced for each of the three stimulus patterns was recorded. Once all of the different designs were recorded, we counted how many times each of the designs was produced, or the design frequency. As mentioned previously, the first three trials of the RFFT contain the same 5-dot matrix. There were instances when designs were produced on one of these trials but then those same designs were not produced on the other trials. Hence, we not only calculated a trial-specific design frequency (i.e., how many times each design was produced on each trial) but also a total design frequency for all of the first three trials combined (i.e., how many times a particular design was produced across all three trials).

## Results

We first sought to characterize the DFC. As mentioned previously, there were a total of 1397 different designs produced across the three different stimulus patterns (Stimulus Pattern A = 656 designs, Stimulus Pattern B = 328 designs, Stimulus Pattern C = 413 designs). Regarding Pattern A, there were anywhere from 1 to 10 lines used to create the different designs and the frequencies ranged from 1 to 121. Designs for Pattern B used anywhere from 1 to 9 lines and the frequencies ranged from 1 to 99. Finally, the designs for Pattern C used from 1 to 10 lines and the frequencies ranged from 1 to 101. Designs comprised a greater number of lines were generally the more infrequently produced designs. Please consult Table 1 for specific information regarding the number of different designs produced for each stimulus pattern.

**Table 1:** Lineage and number of different designs produced for each pattern of the RFFT

	Lineage	Number of different designs
	1 line	10
	2 lines	45
	3 lines	120
	4 lines	168
	5 lines	166
	6 lines	86
	7 lines and up	61
	Total	656
	1 line	10
	2 lines	42
	3 lines	77
	4 lines	98
	5 lines and up	101
	Total	328
	1 line	10
	2 lines	44
	3 lines	94
	4 lines	114
	5 lines	88
	6 lines and up	63
	Total	413
	Grand total	1397

**Fig. 2:**
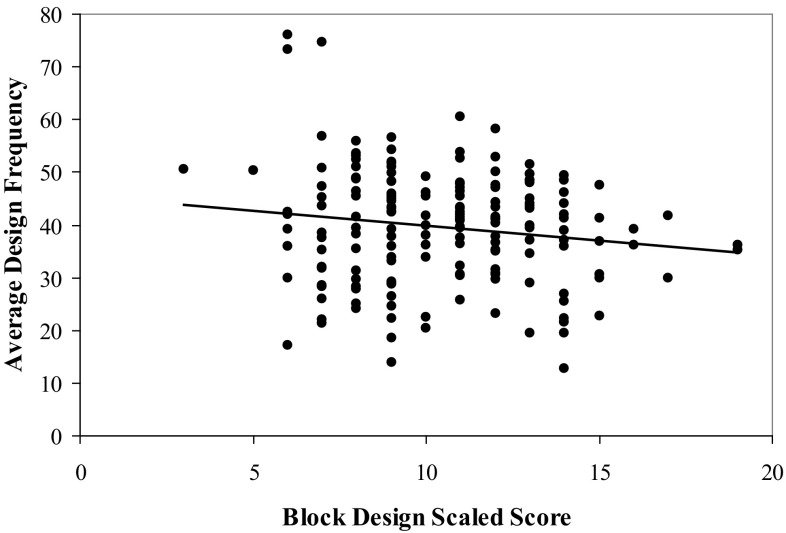
The relationship between Block Design performance and visuospatial spreading activation

We then used the DFC to calculate the average design frequency for each participant. Specifically, for each individual participant, we obtained the frequency for each design produced, using the trial specific design frequencies. The average for each of the five trials was then calculated and then an overall average was calculated from these averages. Hence, an overall average design frequency score was calculated for each of the participants based on the designs they produced. We then conducted correlations between each of the demographic variables of interest and the average design frequency. The results indicated no significant correlations between average design frequency and age (*r* = .09, *p* = .124), education (*r* = −.01, *p* = .460), or Full Scale IQ (*r* = −.08, *p* = .160). We also wanted to know if there were sex differences in regard to average design frequency. The results of a between subjects ANOVA indicated no significant difference, *F*(1, 171) = .88, *p* = .348, between the average design frequency for men (*M* = 40.16, SD = 10.55) and for women (*M* = 38.59, SD = 11.50). Finally, we conducted a series of correlations between performance on each of the neuropsychological tests and average design frequency. The results indicated a significant negative correlation between BD and average design frequency (*r* = −.15, *p* = .028, *R* ^2^ = .02; see Fig. 2). No significant correlations were found between average design frequency and Vocabulary (*r* = −.09, *p* = .122), FTD (*r* = −.08, *p* = .138), FTND (*r* = −.11, *p* = .082), and the total number of unique designs produced on the RFFT (*r* = −.003, *p* = .485).

## Discussion

The results of Experiment I indicated that average design frequency was not significantly related to age, education, or intelligence. Hence, these variables likely do not represent significant confounds that would need to be controlled in subsequent investigations. Further, we felt that since no relationship existed between these variables, there was no need to stratify design frequency by age, education, or intelligence. Importantly, there was also no significant relationship found between the total number of unique designs produced on the RFFT and the average design frequency. This latter finding indicates that fluency and design frequency are measuring different constructs. The findings also indicated that design frequency were related to a test purported to be relatively sensitive to right posterior cerebral functioning and relatively insensitive as a measure of left hemisphere functioning.

These results suggest that design frequency is a separate construct than design fluency and that design frequency is possibly related to right posterior cerebral functioning. However, the results do not necessarily support the proposition that design frequency is a measure of visuospatial spreading activation. As mentioned previously, we have proposed using word frequencies obtained from measures of verbal fluency as a measure of spreading activation in lexical and semantic memory networks. Using the same reasoning, we have proposed that design frequency obtained from a measure of design fluency might then be used as a measure of spreading activation in visuospatial memory networks. Support for using design frequency as a measure of spreading activation might be obtained by examining how design frequency is related to word frequency. Hence, we conducted another study to examine this potential relationship.

## Experiment II

We sought to examine the relationship between verbal spreading activation and visuospatial spreading activation by conducting correlations between design frequency and both word frequencies from the COWAT and from the AN test. Additional exploratory analyses were conducted to examine any potential relationships between design frequency and indices of verbal and nonverbal fluency as well as depression. Our hypothesis was that a significant relationship would exist between design frequency and word frequency, since both are measures of spreading activation, but that the relationship would be relatively weak since one is a measure of visuospatial spreading activation, whereas the other is a measure of verbal spreading activation.

## Methods

### Participants

The sample consisted of 41 undergraduate students (8 men and 33 women) with an age range 18–37 years (*M* = 20.37, SD = 3.41). There were 4 left-handed and 27 right-handed participants. The participants did not have any history of psychiatric hospitalization, chronic drug abuse, or neurological disorder. They were also not taking any psychotropic medications.

### Apparatus

*Animal Naming* The Animal Naming test (AN) test requires the subject to name as many different animals as possible, with no restrictions based on beginning letter or any other characteristic. They are permitted 60 s to generate as many names of animals as possible. The variable of interest included the total number of animal names produced.

Beck Depression Inventory—II. The Beck Depression Inventory—II (BDI-II; [45]) is a 21-item self-report questionnaire used for measuring the severity of depression. The items of the BDI-II address problems related to numerous psychological, cognitive, and physiological symptoms. Each item is rated by the patient on a scale of 0–3, with a range of possible scores from 0 to 63. The raw score was used as the variable of interest.

*Controlled Oral Word Association Test* The Controlled Oral Word Association Test (COWAT) requires the subject to name as many words as possible that begin with a specified letter (F, A, and S) within 60 s. However, they cannot use proper nouns, they cannot count, and they cannot use a stem word and then simply add different endings. The total number of words produced was the variable used.

*Ruff Figural Fluency Test* See description in Experiment I.

### Procedure

This study was approved by the Middle Tennessee State University Institutional Review Board and all participants provided written informed consent. The AN, COWAT, RFFT, and BDI-II were all administered using standard procedures. Following administration of these tests, the frequency of each word generated on the COWAT and the AN test was obtained. We used the Francis–Kucera corpus [46] to obtain the word frequency, recording the frequency for each word generated by the participants. The Francis–Kucera corpus was based on over 1 million graphic words from numerous different sources, including periodicals, novels, newspapers, technical writings, philosophical essays, and writings of fiction. The word frequency for words that violated the rules of the COWAT was not included in the analyses. The average word frequency for the COWAT was calculated by first averaging the word frequencies for each letter used. The average word frequency across the three letters was then calculated, based on the average obtained for each letter. The average word frequency for the AN test was calculated by obtaining the word frequency for each animal named and then averaging across all the names of animals. The average design frequency was then calculated for each participant. Specifically, the frequency of each design produced was obtained and then an average design frequency was calculated for each of the five trials of the RFFT. An overall average was then calculated by averaging across the averages for the five trials.

## Results

**Fig. 3:**
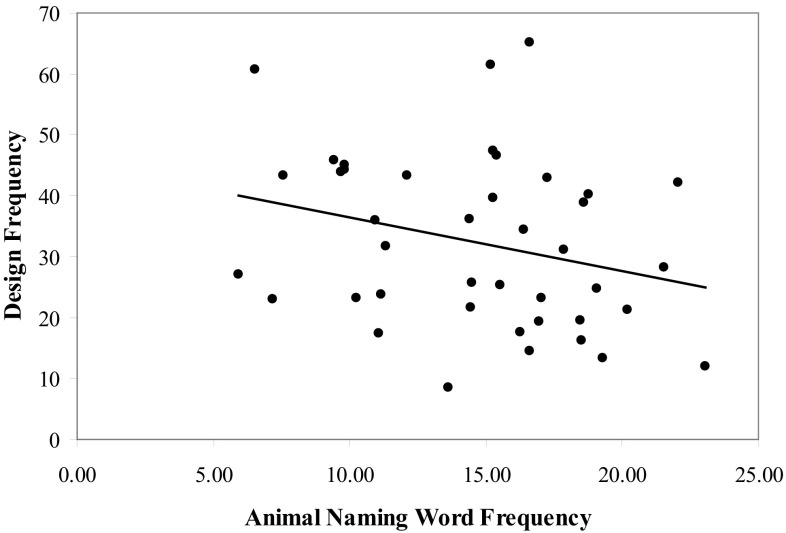
The relationship between verbal and visuospatial spreading activation

Initial correlations were conducted to determine if any significant relationships existed between average design frequency and performance on the COWAT, AN, and BDI-II. We also wanted to determine whether a significant relationship existed between the number of different designs produced on the RFFT and the average design frequency. The results indicated no significant correlation between average design frequency and performance on the COWAT (*r* = .04, *p* = .401), AN (*r* = .003, *p* = .493), or the BDI-II (*r* = −.05, *p* = .377). The relationship between the total number of designs produced on the RFFT and design frequency was also not significant (*r* = .19, *p* = .121). To evaluate our hypothesis, we then conducted correlations between the average design frequency and the average word frequencies from the COWAT and AN test. The results indicated no significant correlation between average design frequency and average word frequency from the COWAT (*r* = .07, *p* = .327). However, a significant negative correlation was found between average design frequency and average word frequency from the AN test (*r* = −.27, *p* = .04, *R* ^2^ = .07; see Fig. 3).

### General discussion

The focus of our first experiment was to describe the creation of the DFC and to determine if our measure of design frequency was significantly related to various demographic variables. The results indicated that design frequency was not related to age, education, intelligence, or sex. We also wanted to determine how design frequency (i.e., visuospatial spreading activation) relates to measures of left and right hemisphere functioning. The findings supported our hypothesis, indicating that design frequency was related to a measure of right posterior functioning (Block Design) but not to a measure frequently found to be relatively sensitive to left posterior functioning (Vocabulary) or to a measure of left frontal (right hand FTT) or right frontal (left hand FTT) functioning. As mentioned previously, evidence exists that supports material-specific memory. Whereas verbal memory has been found to activate the left temporal lobe, nonverbal memory activates the right temporal lobe [47]. Golby et al. [48] reported activation within the left inferior prefrontal cortex and medial temporal lobe during verbal encoding but right inferior prefrontal cortex and right medial temporal lobe activation for pattern encoding. Encoding of words is associated with left dorsal frontal activation and encoding of unfamiliar faces is associated with right dorsal frontal activation [49]. Double dissociation for verbal and nonverbal material has also been demonstrated following left and right thalamic dysfunction [50, 51]. The findings of right hemisphere involvement in visuospatial memory are consistent with the significant correlation observed in our study between design frequency and performance on the Block Design test since research has indicated that performance on this test is related to right posterior functioning [40, 41] and this also supports the proposition that design frequency is related to right posterior cerebral functioning. However, our findings should be considered preliminary in nature. Future research will need to be conducted to provide further evidence for an association between design frequency and right posterior functioning. This evidentiary support might be obtained by correlating design frequency with additional neuropsychological tests purportedly related to right hemisphere functioning. Functional imaging studies might also be used to provide such support.

Although the significant correlation between design frequency and Block Design test performance provides initial support for right hemisphere involvement in design frequency this finding does not necessarily support the proposition that design frequency is a measure of spreading activation in visuospatial memory networks. To support design frequency as a measure of spreading activation, we conducted the second study which involved correlating word frequency, our measure of spreading activation in lexical and semantic memory networks, with design frequency. Our findings supported the hypothesis, indicating the existence of a significant, albeit modest, relationship between average word frequency from the AN test and design frequency. Furthermore, the relationship was negative, suggesting that as spreading activation in visuospatial networks increased spreading activation in semantic memory networks decreased. The existence of a negative relationship between verbal and nonverbal spreading activation is consistent with models of interhemispheric balance. Specifically, Tucker [52] proposed that the left and right cerebral hemispheres exist in a reciprocally balanced relationship, with each hemisphere opposing and complementing the other. This balanced relationship is mediated in part through the corpus callosum, which is involved in both interhemispheric excitation and inhibition [53]. Thus, increased activity in a region of one hemisphere may generate deactivation or decreased activity in the homologous region of the contralateral hemisphere. Research has indicated that semantic fluency tasks, such as performance on the AN test, are associated with left temporal lobe functioning [54, 55]. Given Tucker’s ([52]; see [56]) interhemispheric balance model and the findings of left temporal involvement in semantic fluency, it might be expected that spreading activation within the semantic networks of the left posterior region would be negatively correlated with spreading activation within the visuospatial memory networks of the right posterior region. The integration of these research findings might also explain the lack of a relationship between average word frequency on the COWAT and design frequency since performance on the COWAT is strongly related to left frontal lobe functioning [55, 57–59].

The negative correlation between average AN word frequency and design frequency provides initial support for design frequency being a measure of spreading activation in visuospatial memory networks. However, further research needs to be conducted to provide additional evidence, such as by potentially combining our measure of design frequency with a priming paradigm. Research using lexical decision tasks has indicated that the reaction time for identifying words with higher frequencies is significantly faster than the reaction time for words with lower frequencies [27–29]. The longer reaction time for lower frequency words might suggest that activation of the nodes that represent lower frequency words requires greater spreading activation. Essentially, greater spreading activation is required to activate words that have lower frequencies, i.e., are further out in the lexical/semantic network.

A similar paradigm might be used with design frequency by having participants complete the RFFT and then presenting them with a design-decision task. The stimuli in the task could be comprised designs that they had produced on the test in addition to designs they had not produced. Additionally, the frequency of the designs could be varied such that they are presented with designs that they produced with lower frequencies as well as designs they had produced with higher frequencies. The task would then involve having the participants decide whether or not they had produced designs that are individually presented to them and measuring reaction time. Designs they had previously produced should be associated with quicker reaction times, which would support design frequency as a measure of implicit memory. Additionally, analyses could focus on the effect of design frequency. Specifically, the reaction times should be faster for designs that have higher frequencies as compared to those with lower frequencies, thereby providing support for design frequency as a measure of spreading activation.

Further research might also explore the potential relationship between design frequency, i.e., spreading activation in nonverbal memory networks, and measures of visuospatial episodic memory. Increased spreading activation may facilitate recall of information due to the increased number of retrieval pathways or cues from which the information may be accessed [60]. We conducted a study examining the relationship between performance on a test of supra-span list learning and spreading activation in lexical memory networks as measured by average word frequency of the words produced on the “F” trial of the COWAT. Our findings indicated a significant negative correlation between average word frequency and both immediate and delayed free recall [31, 32]. Hence, as spreading activation increased recall of the word list improved. A similar study could be conducted by examining the relationship between design frequency and performance on measures of nonverbal or visuospatial memory, such as the Rey Complex Figure [61] or the Brief Visuospatial Memory Test—revised [62].

Collins and Loftus [1] proposed that activation within conceptual/semantic networks will decrease over time or with some intervening activity. The mechanisms modulating spreading activation through activation or deactivation of these networks are not completely understood. However, neurotransmitter systems may have a role in modulating spreading activation. For instance, dopamine may act not only as a neurotransmitter but also as a neuromodulator [63], modulating the “signal-to-noise” ratio [64]. Indeed, Kischka et al. [65] reported that dopamine increases the signal-to-noise ratio in semantic networks and results in reduced spreading activation. We have found that Parkinson’s disease, which is associated with a depletion of dopamine, is associated with increased spreading activation in lexical memory networks [33].

The cholinergic system may also have a role in spreading activation. Specifically, patients with dementia who are taking acetylcholinesterase inhibitors exhibit reduced spreading activation in lexical memory networks as compared to dementia patients who were not taking these medications [34]. Research has indicated that dopamine and acetylcholine are asymmetrically distributed in the brain. Kononenko [66] reported greater cholinesterase activity in the left hemisphere, and Jayasundar [67] has reported a greater concentration of choline in left occipital and temporal regions. Others have found greater choline acetyltransferase activity in the left hemisphere [68], and particularly in the left first temporal gyrus [69]. Glick et al. [70] not only found that acetylcholine is predominantly in the left hemisphere but also that greater concentrations of dopamine were found in the left caudate and left globus pallidus. The asymmetrical distribution of dopamine and acetylcholine to the left hemisphere is consistent with their potential role in modulating spreading activation in verbal memory networks. However, given that design frequency may be related to spreading activation within the nonverbal memory networks of the right hemisphere, these same neurotransmitters may have less of a role in modulating nonverbal spreading activation.

Serotonin and norepinephrine, in contrast to dopamine and acetylcholine, seem to be asymmetrically distributed in the right hemisphere. Some have reported greater concentrations of norepinephrine in the right thalamic hemisphere [71]. Regarding serotonin, research has indicated that the right hemisphere has more serotonin metabolite (5HIAA) than the left hemisphere in the medial frontal region [72]. Further, Fink et al. [73] found significantly higher 5-HT1A receptor binding in the superior, middle, and inferior frontal gyri of the right hemisphere. Hence, the possibility exists that serotonin and norepinephrine may have more of a role in modulating spreading activation in nonverbal memory networks. Future research will need to be conducted to examine this possibility.

Limitations of the project include the relative sensitivity of the measures employed to assess left and right cerebral systems via double dissociation technique [74].
